# Integrating fNIRS and machine learning: shedding light on Parkinson's disease detection

**DOI:** 10.17179/excli2024-7151

**Published:** 2024-05-14

**Authors:** Edgar Guevara, Gabriel Solana-Lavalle, Roberto Rosas-Romero

**Affiliations:** 1 CONAHCYT - Universidad Autónoma de San Luis Potosí; 2 Electrical & Computer Engineering Department, Universidad de las Américas-Puebla

**Keywords:** Parkinson's disease, functional near-infrared spectroscopy, machine learning, feature subset selection, genetic algorithms

## Abstract

The purpose of this research is to introduce an approach to assist the diagnosis of Parkinson's disease (PD) by classifying *functional near-infrared spectroscopy *(fNIRS) studies as PD positive or negative. fNIRS is a non-invasive optical signal modality that conveys the brain's hemodynamic response, specifically changes in blood oxygenation in the cerebral cortex; and its potential as a tool to assist PD detection deserves to be explored since it is non-invasive and cost-effective as opposed to other neuroimaging modalities. Besides the integration of fNIRS and machine learning, a contribution of this work is that various approaches were implemented and tested to find the implementation that achieves the highest performance. All the implementations used a logistic regression model for classification. A set of 792 temporal and spectral features were extracted from each participant's fNIRS study. In the two best performing implementations, an ensemble of feature-ranking techniques was used to select a reduced feature subset, which was subsequently reduced with a genetic algorithm. Achieving optimal detection performance, our approach reached 100 % accuracy, precision, and recall, with an F1 score and area under the curve (AUC) of 1, using 14 features. This significantly advances PD diagnosis, highlighting the potential of integrating fNIRS and machine learning for non-invasive PD detection.

## Introduction

Parkinson's disease (PD), a progressive neurodegenerative disorder, is typified by motor symptoms such as rigidity, tremors, and bradykinesia (Varadi, 2020[23]). The integration of neuroimaging techniques such as Positron Emission Tomography (PET) and functional Magnetic Resonance Imaging (fMRI) with machine learning has significantly advanced research in PD, offering profound insights into the disease's neural mechanisms. Functional imaging studies have provided powerful tools to study the functional anatomy and pathophysiology of PD, enabling the analysis of task-specific changes in regional cerebral blood flow and blood oxygenation level dependent (BOLD) effects (Ceballos-Baumann, 2003[3]). Resting State fMRI (RS-fMRI) has been utilized to identify frequency-specific changes in resting brain activity, offering a novel perspective in PD diagnosis through machine learning approaches (Tian et al., 2020[22]). Additionally, advancements in data analysis methods using Empirical Mode Decomposition (EMD) have been explored to investigate temporal changes in early PD, further emphasizing the role of neuroimaging in understanding the disease's progression (Cordes et al., 2018[5]). These studies underscore the potential of combining advanced neuroimaging with machine learning to enhance the diagnosis, understanding, and treatment of PD, paving the way for more effective interventions and improved patient outcomes. In summary, neuroimaging coupled with machine learning techniques is a promising approach for detecting PD potentially leading to earlier diagnosis and more personalized treatment strategies.

Functional Near-Infrared Spectroscopy (fNIRS) is a non-invasive optical imaging technique. It measures the hemodynamic responses associated with neural activity, specifically monitoring the changes in blood oxygenation and blood volume in the cerebral cortex. fNIRS utilizes near-infrared light to penetrate the skull and measure the differential absorption of light by oxy-hemoglobin and deoxy-hemoglobin, thus providing insights into brain function. This method is valuable due to its safety, non-invasiveness, relative low-cost, portability, and ability to provide measurements of the brain's hemodynamic changes linked to neuronal activity (Ayaz et al., 2022[1]). Yet, the potential of fNIRS as a diagnostic tool for PD remains largely unexplored. While ML techniques have been widely applied to other neuroimaging modalities like MRI, PET and electroencephalography (EEG) in PD research (Desai, 2023[6]; Thummikarat and Chongstitvatana, 2021[21]), the integration of fNIRS data in these studies is less common. 

The application of fNIRS combined with machine learning techniques in the study of PD illustrates a promising frontier for diagnosing and understanding the neurofunctional correlates of this neurodegenerative disorder. Research has shown the effectiveness of hybrid EEG-fNIRS models and machine learning algorithms in classifying diseases and assessing brain function, particularly in movement-related tasks significant to PD. There are studies that demonstrate the potential of fNIRS to provide insights into the prefrontal cortex's role in motor function, suggesting its utility in exploring PD's impact (Cicalese et al., 2020[4]; Nieuwhof et al., 2016[17]). Furthermore, advancements in machine learning have enhanced the analysis of fNIRS data, offering frameworks to distinguish PD patients from healthy controls (Hamid et al., 2022[8]). Additionally, the exploration of functional degeneration through fNIRS-based brain state transitions and connectivity analysis underscores the technique's capability to capture neurofunctional alterations associated with PD (Lu et al., 2022[15]). Collectively, these studies underscore the valuable contributions of fNIRS and machine learning in advancing PD diagnosis and understanding, marking a step forward in the non-invasive exploration of brain activity and functional changes inherent to the disease.

fNIRS could provide unique insights into the cerebral hemodynamics associated with PD, offering a non-invasive, cost-effective alternative to traditional neuroimaging methods. The objective of this study is to integrate functional Near-Infrared Spectroscopy (fNIRS) with ML for PD detection. Our focus is on identifying the most effective feature selection methods and ML algorithms suitable for this purpose.

The “Materials and Methods” section discusses fNIRS and ML are applied to the task of PD detection. The “Results and Discussion” section provides a discussion of the experimental results obtained by following different strategies of feature subset selection. 

## Materials and Methods

**Figure 1:**
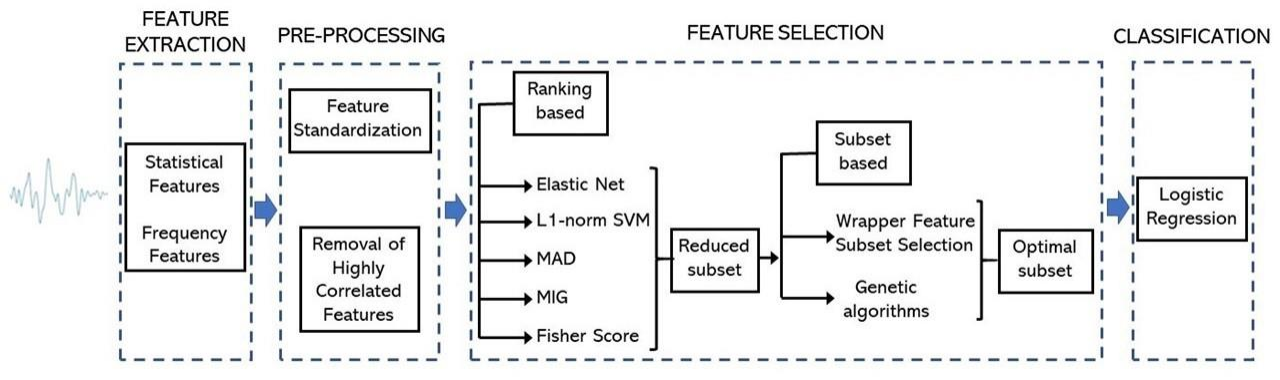
Methodology for PD detection by analyzing fNIRS signals from participants. The methodology consists of four states: feature extraction, feature pre-processing, feature selection and feature classification.

An overview of the proposed approach for PD analysis on fNIRS studies is shown in Figure 1(Fig. 1). This approach consists of four stages: (1) feature extraction from each fNIRS study, (2) standardization of extracted features, (3) selection of the most relevant features and subsequent selection of those relevant features with the best classification performance with Wrapper Feature Subset Selection (WFSS) and a genetic algorithm, (4) classification to identify an individual as having PD or not. This methodology was implemented in Python version 3.10.9.

### Data set

The data set acquisition was conducted on recruited participants with signed informed consents according to the Declaration of Helsinki (with registration number 77-21) at the Neurology Department of the Central Hospital “Dr. Ignacio Morones Prieto” in Mexico from October 2021 to October 2022. The participants were seated with closed eyes as motionless as possible during six minutes under low lighting so that they were relaxed and without fallen asleep. Twenty PD patients were enrolled and diagnosed by a neurologist according to the criteria of the United Kingdom PD Society Brain Bank (Hughes et al., 1992[10]), where the degree of disease severity was determined using the Hoehn and Yahr (HY) scale (Hoehn and Yahr, 1967[9]) and the Movement Disorder Society-Sponsored Revision of the Unified Parkinson Disease Rating Scale (MDS-UPDRS, part III) (Goetz et al., 2007[7]), and these ratings were validated by an independent rater (IRL). All evaluations were conducted while the PD patients were not taking anti-parkinsonian medication, approximately 12 hours after the last dose. The control group was composed of twenty individuals matched in terms of age and sex, whose cognitive functions were assessed with the Montreal Cognitive Assessment (MoCA) (Nasreddine et al., 2005[16]), where severe cognitive impairment was established at a score bellow 10 on the MoCA examination. 

**Figure 2:**
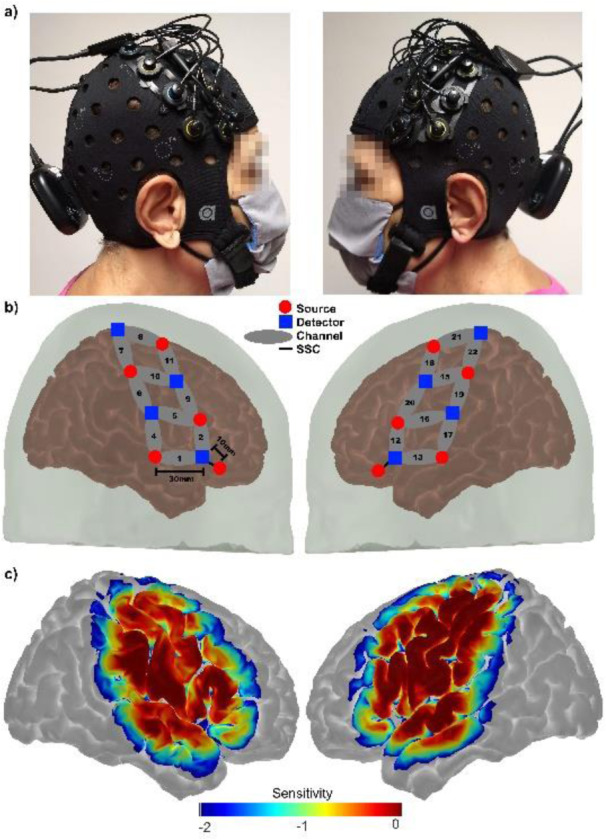
Figure 2: a) Pictures showing the location of an optode array on a single participant's skull. b) Registered probe geometry: the eight detectors are designated by blue squares, while the ten sources are indicated by red circles. Long separation channels (30 mm) are indicated by black numerals in gray ellipses, and short separation channels (SSC = 10 mm) are indicated by black lines, c) a logarithmic temperature plot representing the probe's sensitivity to detect brain hemodynamics goes from 1.00 (0 dB, red) to 0.01 (-40 dB, blue) times the maximum sensitivity.

**Figure 3:**
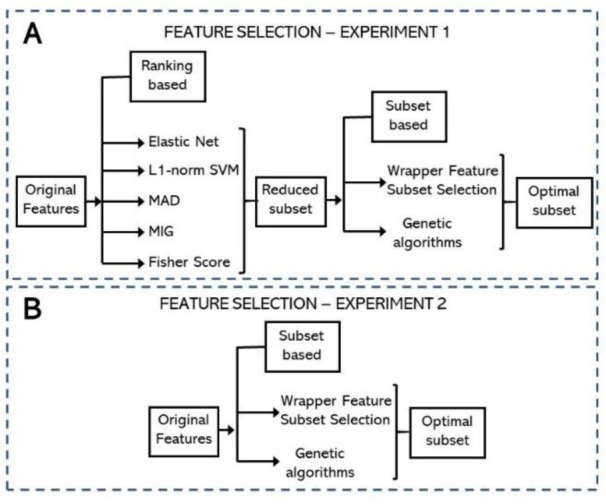
Two settings for feature selection. A) The first setting consisted of an ensemble of feature ranking techniques, followed by feature subset selection using WFSS or GA. B) Feature subset selection (WFSS or GA), without feature ranking, was used in the second setting.

Signals carrying brain activity were recorded with the portable fNIRS system Brite MKII (Artinis Medical Systems BV, the Netherlands), depicted in Figure 2a(Fig. 2). This system consists of (1) ten dual-wavelength LEDs centered at 757 and 843 nm at a sampling frequency of 25 Hz (red circles in Figure 2b(Fig. 2)), (2) eight detectors (blue squares in Figure 2b(Fig. 2)), (3) twenty long channels (10 per hemisphere) to measure hemoglobin changes across bilateral motor brain regions (Figure 2c(Fig. 2)), and (4) two short channels to minimize the effects of superficial hemodynamics (Brigadoi and Cooper, 2015[2]; Tachtsidis and Scholkmann, 2016[20]). For each participant at resting state, 22 fNIRS signals were recorded during six minutes at a sampling frequency of 25 Hz, and each signal was converted into three absorption contrasts HbR (deoxyhemoglobin), HbO (oxyhemoglobin), and HbT (total hemoglobin). Care was taken to remove channels with poor signal and correct motion artifacts via spline and wavelet decomposition (Novi et al., 2020[18]).

### Feature extraction and pre-processing

Channels with poor signals were eliminated and motion artifacts were corrected. A total of 792 features were extracted from each participant, and these features were categorized into two groups: 396 temporal features and 396 spectral features. 

The total number of temporal features per participant was 22 channels × 3 bands per channel × 6 characteristics per band = 396 statistical features per participant. Six temporal features were calculated from each band (HbO, HbT, HbR): (1) maximum absolute value


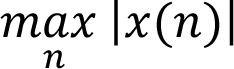
,

(2) average value


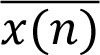
,

(3) variance


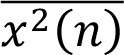
,

(4) skewness


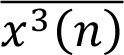
,

(5) average time between two consecutive zero crossings, (6) average time between a zero crossing and a peak value. An analysis of the frequency spectrum of each band *X*(ω) was conducted to extract six spectral features: (1) average value, (2) variance, (3) skewness, (4) kurtosis, (5) energy, (6) dominant frequency.

### Feature selection

The number of extracted features per participant was high. Instead of using one feature selection method, ensemble feature selection was used to reduce the number of features and the complexity of the classification model (Jong et al., 2004[12]; Jiménez et al., 2022[11]). An ensemble of methods combines the strengths of various methods to obtain an optimal feature subset and enhance classification performance. Two categories of feature selection methods were used: *feature ranking* (MAD filter, MIG filter, Fisher's score, elastic net regression, L1-norm SVM) followed by *feature subset selection*: Wrapper feature subset selection (Kohavi and John, 1997[14]), Genetic algorithms (Katoch et al., 2021[13]). 

In one set of experiments, those features that were ranked by at least two filters (MAD, MIG, Fisher's score) in the top 1 % were selected to train a logistic classifier and measure its accuracy. This process was repeated for those features that were ranked in the top 2 % and up to the 10 %, which resulted in ten feature subsets. The feature subset with the highest accuracy was selected. The L1-norm SVM and elastic net regression methods were also run to generate two corresponding feature subsets. Finally, the three subsets obtained by running three selecting strategies (filters, L1-norm SVM, elastic net regression) were combined.

In a second set of experiments, feature subset selection was conducted by using Wrappers feature subset selection (WFSS) or Genetic Algorithms (GA).

#### Mean absolute difference (MAD) filter

This filtering method selects features irrespective of the used classifier. Each feature is evaluated individually. If a feature has zero mean absolute difference from the mean value, then it is removed. It is assumed that features with higher mean absolute difference are likely to contain more information.

#### Mutual information gain (MIG) filter

This method evaluates the information gain for each feature, and those features that maximize the information gain are selected. The selected features in turn minimize the information entropy so that the separation of the corresponding classes is the most effective for discrimination.

#### Fisher's score feature selection

The Fisher score feature selection ranks features based on their discriminative power in a dataset with various classes. The Fisher's score is the ratio of the between-class variance and the within-class variance. A feature with high Fisher's score is more discriminative for classification.

#### Elastic net regression

The *elastic net regression* performs feature selection by shrinking the coefficients of irrelevant features to zero, which results in a model with a reduced feature subset. The *elastic net regression* combines the strengths of (1) the Lasso regression that adds an L1 penalty term to encourage sparsity in the feature set and (2) the Ridge regression that adds an L2 penalty term to shrink less relevant features towards zero.

#### L1-norm SVM for feature selection

The SVM uses a kernel function (linear, polynomial, or radial basis function) to learn decision hyper-planes and by tuning the hyperparameters embedded feature selection can be performed. The model was trained with a technique that *constrains *or *regularizes *the coefficients with the effect of forcing some of the parameter estimates to be zero so that the corresponding features are discarded.

#### Wrappers feature subset selection (WFSS)

In this method various feature subsets are obtained, then a classifier is trained with each feature subset, and the classifier with the best performance corresponds to the selected feature subset. WFSS is essentially a search problem. Instead of greedily generating all the possible feature subsets, which is computationally very expensive, heuristics are used to generate a reduced number of feature subsets (Kohavi and John, 1997[14]). Forward selection is an iterative heuristic, which keeps adding the feature which best improves a classifier. Backward elimination removes the least significant feature at each iteration in terms of classification performance. WFSS heuristics iteratively keeps aside the best or the worst performing feature at each iteration. It then ranks the features based on the order of their elimination.

#### Feature selection based on genetic algorithms

Genetic algorithms select a feature subset based on evolution (Katoch et al., 2021[13]). Each feature subset is represented with a binary code in which an absent feature is coded as zero while a present one is coded as one. The first step is to randomly generate a collection of binary codes that corresponds to a population of feature subsets. Each member of the population is evaluated using a classifier. According to their performance it is determined which subsets will give rise to the next generation, where each member is the result of crossing over two codes from two winners of the previous generation (parents). Besides cross over, mutation randomly introduces or removes some features from each child. This process leads to an improved classification performance overall as the weaker feature subsets are progressively eliminated.

### Classification

Features are extracted from fNIRS signals, the most relevant features are selected and they are classified with a logistic regression model. Logistic Regression (Schein and Ungar, 2007[19]) is a classification technique that uses a logistic function to model the relationship between the features extracted from a signal and the dependent variable, which corresponds to two possible classes, either PD is present or absent. The logistic regression maps feature vectors to probabilities using a sigmoid function with values between 0 to 1. The sigmoid function is differentiable which allows the minimization of a cost function during learning of the logistic regression model.

### Performance assessment

After feature subset selection, k-fold cross validation was used to evaluate classification performance. A set of feature vectors was partitioned into k folds, k −1 folds were used to train the logistic classifier, and the remaining fold was used to measure classification performance. The process was repeated k times so that the classification performance was evaluated with each fold. Finally, the global performance metrics were obtained by averaging the metrics at each fold. For this work, k = 5 and 10. During evaluation of a PD detection method, if a PD patient is correctly identified then this case is a TRUE POSITIVE (TP); otherwise, it is a FALSE NEGATIVE (FN). Healthy participants, correctly identified, correspond to TRUE NEGATIVES (TN); otherwise, they are FALSE POSITIVES (FP). To measure PD detection performance, accuracy, precision, recall, F1 score and the area under the curve (AUC) are used as metrics. Accuracy is the percentage of classification results that are correct, *Accuracy* = (*TP+TN*) / (*TP+FN+TN+FP*). Recall is the probability that PD detection is positive given that the participants are PD patients, *recall* = *TP* / (*TP+FN*). Precision is the percentage of PD cases detected as positives, *Precision* = *TP* / (*TP+FP*). The F1 score is the harmonic mean of precision and recall, *F*1 = 2 / (1/*recall*+1/*precision*) = 2*TP* / (2*TP+FP+FN*).

## Results and Discussion

Figure 3(Fig. 3) describes the settings of two experiments to assess the performance of the proposed PD detection approach based on fNIRS analysis. These experiments are different in terms of the implementation of feature selection. In a first set of experiments, the number of extracted features (792 features) was reduced using (1) an ensemble of feature ranking strategies, followed by (2) feature subset selection strategy based on one of two strategies, WFSS or GA (Figure 3A(Fig. 3)). A second set of experiments consisted of reducing the 792 extracted features by only using a feature subset selection strategy, either WFSS or GA (Figure 3B(Fig. 3)).

At each experimental setting, the logistic regression model was used for classification. The top half of Table 1(Tab. 1) shows PD detection performance assessment with 10-fold cross-validation after conducting the first series of experiments. In this setting, feature selection was accomplished by first applying an ensemble of feature ranking techniques (elastic net, L1-norm SVM, MAD, MIG, Fisher score), followed by further narrowing down the top-rated features by running a feature subset selection technique along with the logistic regression. The selected feature subset by these two stages is used to evaluate PD detection performance with 10-fold Cross-Validation. The second stage that reduces the set of the top-rated features was implemented in two ways, either with WFSS or with GA. It is observed that the performance of PD detection based on GA (third row) is higher than the performance of PD detection based on WFSS (second row). The bottom half of Table 1(Tab. 1) shows the results of assessing the PD detection performance when the feature subset selection (WFSS or GA) was conducted on the entire set of extracted features without the use of the ensemble of ranking techniques for pre-selection of a feature subset.

The implementation of PD detection according to the first setting (top half of Table 1(Tab. 1)) is characterized by another advantage in terms of model complexity since the number of selected features is smaller than that corresponding to the second setting (bottom half). In addition, it is observed that the performance of PD detection based on GA (sixth row) is higher than that of PD detection based on WFSS (fifth row). The results in Table 1(Tab. 1) also show that the implementation of feature subset selection with a genetic algorithm corresponds to higher PD detection performance. An initial generation with 1000 subsets (solutions) was used to run 100 iterations of the genetic algorithm, i. e., 100 generations of solutions were produced. At each generation, new solutions were born as a result of using a cross-over probability of 0.05 and a mutation probability of 0.03. Accuracy was the fitness function used as criterion to select the best solutions in a new generation.

Table 2(Tab. 2) shows the performance assessment of the proposed PD detection approach with 5-fold cross-validation. The top half contains the results that were obtained with the first experimental setting, where feature selection was implemented with (1) an ensemble of feature ranking techniques, followed by (2) feature subset selection. Feature subset selection was implemented with WFSS (second row) or GA (third row). The bottom half shows the result obtained with the second experimental setting, where feature selection was implemented with feature subset selection without any previous feature ranking technique.

According to the results reported in Table 2(Tab. 2), the first experimental setting (second and third rows) presented higher PD detection performance than the second experimental setting (fifth and sixth rows). The implementation of feature subset selection with a genetic algorithm (third and sixth rows) is characterized by the best PD detection performance. PD detection is accurate when it is implemented with an ensemble of feature ranking techniques followed by feature subset selection based on a genetic algorithm. The genetic algorithm was run 100 times, i. e., 100 generations were obtained, where each generation consisted of 1000 solutions (feature subsets) produced with a cross-over probability of 0.05, a mutation probability of 0.03, and PD detection accuracy was used as criterion to select the fittest solutions from each generation. The last column of Table 2(Tab. 2) reports the number of selected features for each PD detection implementation.

## Conclusion

In conclusion, we integrate functional Near-Infrared Spectroscopy (fNIRS) with machine learning for detection of Parkinson's Disease by testing various implementations. Each setting starts with a feature set of high dimensionality (792 features), where two groups of features were used, statistical and spectral. At each setting, a combination of feature selection strategies is used to estimate the most relevant feature subset. Accurate PD detection is achieved when an ensemble of feature ranking strategies is used to select a feature subset, which is further reduced with a genetic algorithm, resulting in an accuracy of 1, precision of 1, recall of 1, F1 score of 1 and AUC of 1. Accurate detection performance was obtained by feeding a logistic classifier with 14 features (5-fold cross-validation) and 16 features (10-fold cross-validation).

Our approach, with remarkable detection accuracy, significantly advances PD diagnosis, showcasing the potential of combining fNIRS and machine learning for efficient, non-invasive PD detection.

## Declaration

### Data availability statement 

The data set that contains functional near-infrared spectroscopy (fNIRS) data from twenty PD patients and twenty healthy individuals is available via Zenodo: https://zenodo.org/records/7966830. There are three folders, each corresponding to a different task: twenty trials of a ten-second finger-tapping task, a two-minute walking task, and the task under analysis in this work, six-minute resting state.

The code to analyze fNIRS signals with machine learning to assist PD diagnosis is available in GitHub: https://github.com/GabrielSolana29/rs_fNIRS_PD.git. This link describes the steps to execute the code.

### Ethics statement

The studies were conducted on human participants who signed informed consent according to the Declaration of Helsinki (with registration number 77-21) at the Neurology Department of the Central Hospital “Dr. Ignacio Morones Prieto” in Mexico from October 2021 to October 2022.

### Authors' contributions 

Edgar Guevara contributed to data collection. All authors were involved in statistical analyses, data interpretation, and manuscript. All authors planned and designed the study. All authors read and approved the final manuscripts. 

### Acknowledgments 

We would like to thank Ildefonso Rodríguez-Leyva for providing the patients' clinical data and Francisco Javier Rivas-Ruvalcaba for his assistance in some of the recordings.

### Conflict of interest 

The authors declare that they have no conflict of interest. 

### Funding 

This work was supported in part (E. G.) by the "IxM CONAHCYT" program, project 528, and "Frontier Science" project 20884. 

## Figures and Tables

**Table 1:**
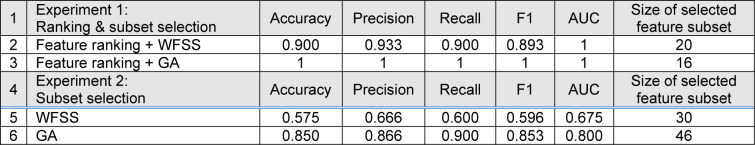
Assessment evaluation of the proposed PD detection approach using 10-fold cross-validation. The top half of the Table shows the detection performance for the first set of experiments where an ensemble of feature-ranking techniques was followed by feature subset selection implemented with WFSS (second row) or with GA (third row). The bottom half of the Table shows the detection performance for the second set of experiments where feature subset selection was only used with a WFSS implementation (fifth row) or GA (sixth row). The last column shows the size of the selected feature subset for each implementation.

**Table 2:**
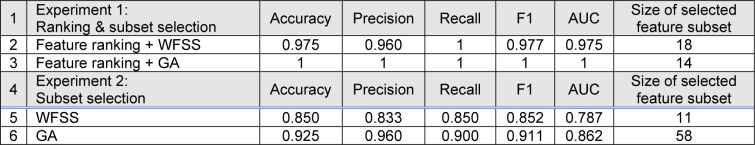
Assessment evaluation of the proposed PD detection approach using 5-fold cross-validation. The top half of the Table shows the detection performance for the first set of experiments where an ensemble of feature-ranking techniques was followed by feature subset selection implemented with WFSS (second row) or with GA (third row). The bottom half of the Table shows the detection performance for the second set of experiments where feature subset selection was only used with a WFSS implementation (fifth row) or GA (sixth row). The last column shows the size of the selected feature subset for each implementation.
